# Clinically integrated multi-organ point-of-care ultrasound for undifferentiated respiratory difficulty, chest pain, or shock: a critical analytic review

**DOI:** 10.1186/s40560-016-0172-1

**Published:** 2016-08-15

**Authors:** Young-Rock Ha, Hong-Chuen Toh

**Affiliations:** 1 Emergency Department, Bundang Jesaeng Hospital, 20 Seohyeon-ro 180beongil, Bundang-gu, Seongnam-si, Gyeonggi-do South Korea; 2 Acute and Emergency Care Centre, Khoo Teck Puat Hospital, 90 Yishun Central, S768828 Singapore, Singapore

**Keywords:** Multi-organ point-of-care ultrasound, Respiratory difficulty, Chest pain, Shock

## Abstract

Rapid and accurate diagnosis and treatment are paramount in the management of the critically ill. Critical care ultrasound has been widely used as an adjunct to standard clinical examination, an invaluable extension of physical examination to guide clinical decision-making at bedside. Recently, there is growing interest in the use of multi-organ point-of-care ultrasound (MOPOCUS) for the management of the critically ill, especially in the early phase of resuscitation. This article will review the role and utility of symptom-based and sign-oriented MOPOCUS in patients with undifferentiated respiratory difficulty, chest pain, or shock and how it can be performed in a timely, effective, and efficient manner.

## Background

The capability to recognize and resuscitate the critically ill, or peri-cardiac arrest patients, is one of the defining traits of critical care and emergency medicine. These patients can be categorized into three groups: pre-arrest, intra-arrest, and post-arrest with return of spontaneous circulation (ROSC). For all three groups, and especially the pre-arrest patients, rapid diagnosis of the underlying physiology and etiology and timely intervention are essential for effective management and stabilization. Speedy and accurate clinical decisions can be lifesaving. Traditionally, acute care physicians evaluate patients based on history and physical examinations. For those presenting with respiratory difficulty, chest pain, shock, or shock-related symptoms or signs, the assessment has to be performed in a focused and time-sensitive manner. Now, bedside multi-organ point-of-care ultrasound (MOPOCUS) and MOPOCUS-guided protocols can be used as an adjunct to standard clinical examination, especially during the initial and undifferentiated phase. MOPOCUS can provide many critical pieces of information to guide clinical decision-making, while waiting for laboratory and imaging results.

According to the consensus statement of the American Society of Echocardiography and the American College of Emergency Medicine [1], respiratory difficulty, chest pain, or shock are recommended indications of the focused cardiac ultrasound in an emergency setting. A growing body of evidence also supports the use of MOPOCUS of the critically ill to evaluate cause of shock or dyspnea [2–15]. Although there are only few studies reporting the utility of MOPOCUS using chest pain alone as the primary indication, the astute clinician is cognizant that etiologies classically associated with chest pain, such as acute coronary syndrome and aortic dissection, can be associated with dyspnea or hypotension or even presents atypically with these two “non-cardiac” presentations alone in the absence of chest pain. A patient with pneumothorax can present with shortness of breath and chest pain and develop hypotension when it becomes a tension pneumothorax. Acute myocardial infarction complicated with cardiogenic shock and pulmonary edema can produce dyspnea, chest pain, and shock concurrently. Indeed, the patient’s signs and symptoms can vary depending on the severity of disease and presence of complications. Therefore, it is prudent for acute care physicians to perform a symptom- or sign-based MOPOCUS for any combination of the three indications listed above.

MOPOCUS is a powerful adjunct to clinical assessment. The certainty of presumptive diagnosis derived from history-taking and physical examination can be validated, or occasionally refuted, by information provided by MOPOCUS. In this article, we will appraise the utility of an integrated MOPOCUS, focusing on the differential diagnostic process in pre-cardiac arrest situation and the sequence of scanning. A detailed review of each organ, especially the abdomen, using point-of-care ultrasound (POCUS) will be covered subsequently in this thematic series.

### The sequence of MOPOCUS scanning

**Fig. 1:**
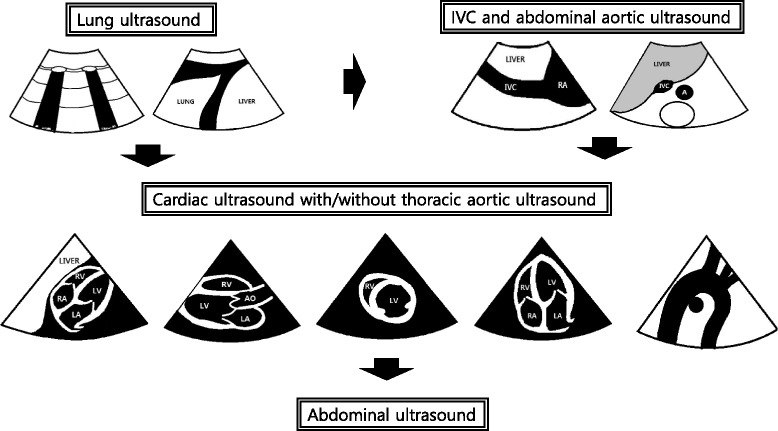
Sequence of MOPOCUS scanning

**Fig. 2:**
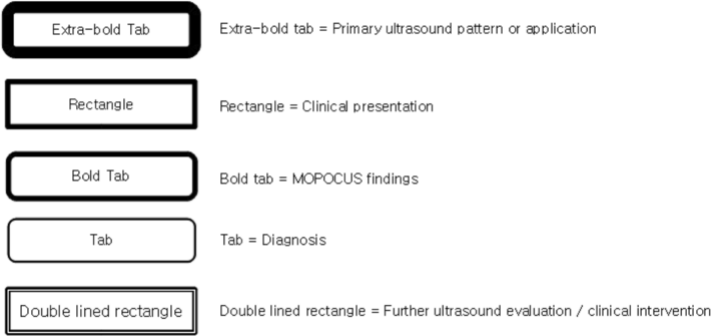
The algorithm (Figs. 2, 7, 8, 12, 14, 16, and 19) begins at the top with the primary ultrasound finding or application (*extra bold tab*) and primary clinical presentation (*rectangle*) and proceeds downwards. The specific MOPOCUS findings are indicated by the *bold tab*, while the *tab* itself represents the diagnosis. The *double-lined rectangular frame* suggests further ultrasound assessment or clinical intervention. The sequence of assessment and interpretation is guided by the *black line* behind these *icons*

There is no universally accepted sequence of scanning using MOPOCUS. In this review, we advocate that the physician begin by assessing the lung and inferior vena cava (IVC), with the abdominal aorta, followed by the heart (including the thoracic aorta in case of chest pain) and, lastly, the abdomen for evaluation of the source of intra-abdominal sepsis or blood loss (Fig. 1). Although all the organs can be scanned with either an abdominal convex (2–6 MHz) or cardiac sector (2–4 MHz) transducer, we can change the transducers for a detailed evaluation if time permits. This sequence is both practical and time-efficient. Firstly, ultrasound findings from the lung and IVC allow rapid categorization of the causes of dyspnea or shock. Secondly, the critically ill are most often supine, a position that is conducive for scanning these two systems. Lastly, from the same site for IVC evaluation, the physician can easily tilt the probe into the subxiphoid plane to evaluate the heart and integrate the focused cardiac ultrasound findings with those from the lung and IVC to elucidate the pathophysiology of shock. In this review, we formulated these MOPOCUS findings into several structured algorithmic approaches. While these are not exhaustive, the underlying pathophysiology and hemodynamics can be systematically categorized and subsequently narrowed to those that are critical, commonly encountered, and warrant timely diagnosis and intervention. The legends used in the algorithms (Fig. 2, 7, 8, 12, 14, 16, and 19) are detailed in Fig. 2.

### Lung ultrasound

The first and most important ultrasound sign to recognize in the lung is the “bat sign.” The bat sign is essential for the accurate identification of the pleural line. Conceptually, the lung should be interrogated in three zones: the chest wall, pleural line, and subpleural space. Sonographic findings and their definitions at each part are summarized in Table 1.

**Table 1:** Interpretation of lung ultrasound

Location	Normal findings	Abnormal findings
Chest wall	Hypoechoic intercostal muscle and echoic ribs with acoustic shadow	Subcutaneous emphysema (E-lines)
Pleural line	Lung sliding	Lung point
	Lung pulse	Pleural line abnormalities
		• Irregular
		• Thickened
		• Fragmented
Supleural space	A-lines^a^	Multiple B-lines (3 or more per intercostal space) consolidation pleural effusion
	Few or no B-lines (2 or less per intercostal space)	

^a^A-lines can also be seen in pathologic situation, such as a pneumothorax, though without lung sliding in this case

Do we need to scan the entire lung when performing lung ultrasound? On the one hand, in the interest of rapid assessment, many favor the BLUE protocol described by Dr. Lichtenstein which uses only three points on each chest [16]. Some sampled five to seven points, taken to be representative of the areas covered [12, 17]. In the comprehensive lung ultrasound, all intercostal spaces are scanned. Regardless of the number of sites scanned, five sonographic lung patterns can be distinguished: normal lung pattern, pneumothorax, interstitial syndrome, alveolar consolidation, and pleural effusion. For practical purposes, we can categorize them into “non-diffuse interstitial pattern” (subdivided into normal lung pattern and abnormal non-diffuse interstitial pattern) and “diffuse interstitial pattern.” This review will describe these lung patterns in the context of different clinical situations and integrate them using the concept of MOPOCUS.

### Normal lung pattern

**Fig. 3:**
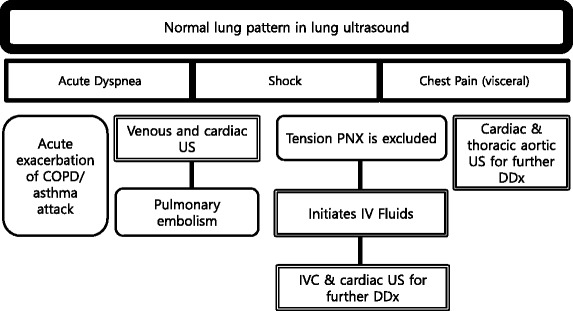
Algorithm for normal lung pattern in lung ultrasound. *COPD* chronic obstructive lung disease, *US* ultrasound, *PNX* pneumothorax, *DDx* differential diagnosis

**Fig. 4:**
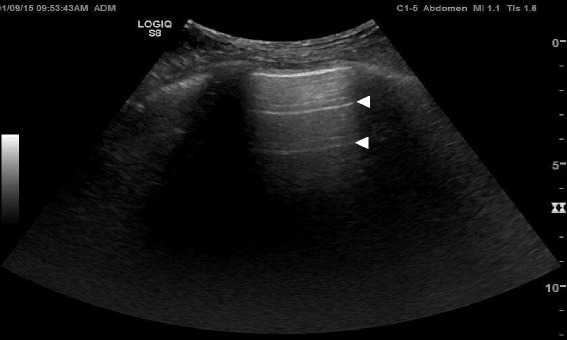
A-lines. A-lines (*arrowheads*) are horizontal artifacts generated by the repeated reflection of the ultrasound beam between the pleural line and the probe surface

Normal lung pattern is defined as A-lines with the lung sliding on the anterolateral chest examination bilaterally, without alveolar consolidation or pleural effusion on posterior examination (Figs. 3 and 4). It is important to recognize that a normal lung pattern does not equate a normal lung. Acute dyspnea and a normal lung pattern can be seen in acute exacerbation of chronic obstructive pulmonary disease (COPD) or asthma attack [16]. Pulmonary embolism (PE) can also have normal lung ultrasound findings, especially in the absence of peripheral lung infarction. In a recent systematic review, the accuracy of lung ultrasound alone to detect PE has an estimated sensitivity of 87.0 % and a specificity of 81.8 % [18]. Lichtenstein added a venous analysis right after identifying an A-pattern on anterior chest examination: the A-pattern plus deep vein thrombosis in the venous analysis has a sensitivity of 81 % and a specificity of 99 % for PE [16, 19]. Nazerian et al. reported that MOPOCUS yielded a sensitivity of 90 % and a specificity of 86.2 % for the diagnosis of PE, comparing that with respective test characteristics of isolated system evaluation: lung ultrasound (60.9 and 95.9 %), cardiac ultrasound (32.7 and 90.9 %), and venous analysis (52.7 and 97.6 %) [20]. This supports the rationale of using an integrated, rather than isolated, approach when performing POCUS.

A normal lung pattern in patients with shock warrants two immediate follow-up actions: the first is to rule out tension pneumothorax and, secondly, to initiate fluid resuscitation based on the Fluid Administration Limited by Lung Sonography (FALLS) protocol [21–23]. Although it has not been validated in shock, non-diffuse interstitial pattern in critically ill patients had a 97 % positive predictive value for a pulmonary artery occlusion pressure of 18 mmHg or less [24]. Apart from tension pneumothorax, a caval and cardiac ultrasound following lung examination will help define the remaining causes of obstructive shock.

The last pearl to note is that chest pain in patients with a normal lung pattern is mostly visceral in nature. The physician should focus the search for the etiology using cardiac and aortic ultrasound.

### Pleural diseases

#### Pneumothorax

**Fig. 5:**
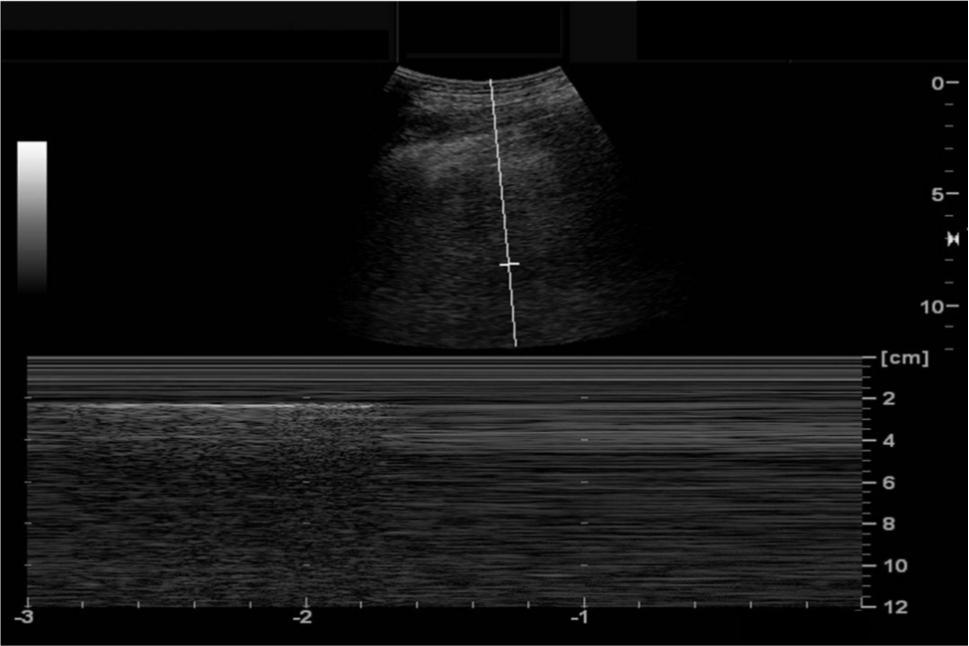
Lung point. Alternating seashore sign (*left*) and stratosphere sign (*right*) on M mode is pathognomonic for pneumothorax

Patients with pneumothorax present with shortness of breath and pleuritic chest pain. The absence of lung sliding does not have adequate specificity to rule in the disease, as this absence can be observed in severe emphysema, adult respiratory distress syndrome (ARDS), and atelectasis [25, 26]. The lung point is highly specific for and thus rules in pneumothorax (Fig. 5) [27]. The presence of lung sliding, B-line, or lung pulse rules out pneumothorax, as all of them require the apposition of the parietal and visceral pleura [21].

When the size of the pneumothorax becomes large enough to surround the entire lung surface, the lung point will disappear. Consequently, the acute care physician should not waste time looking for the lung point and thus delay a chest tube insertion, especially when the patient is in shock. In this case, one would expect to find a plethoric IVC on the subxiphoid view, with the heart displaced to the contralateral side. Tension pneumothorax is the first etiology to rule out among the other causes of obstructive shocks.

#### Pleural effusion

**Fig. 6:**
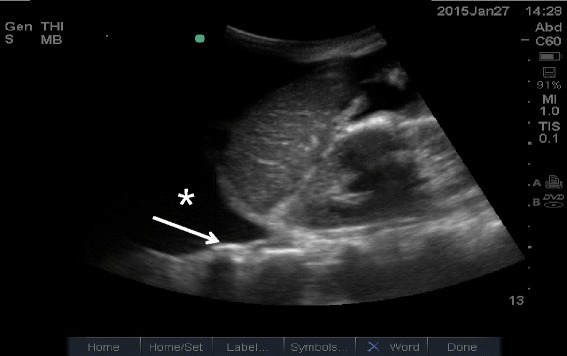
Pleural effusion. Pleural effusion (*asterisk*) permits the ultrasound beam to penetrate deeply to reveal the vertebral stripe (*arrow*). The vertebral stripe will not be visible above the diaphragm if the lung is aerated

Pleural effusion can be identified in posterolateral lung examination (Fig. 6). It can cause respiratory difficulty, pleural chest pain, or both. The amount and nature of pleural effusion can be estimated by using an inter-pleural distance or area and sonographic appearances [28–30].

A large pleural effusion can cause respiratory embarrassment, hypovolemic shock (especially in a large hemothorax), or even obstructive shock due to compression of the IVC and heart, which induces the diastolic failure [31]. In patients who required mechanical ventilation and had a significant transudate pleural effusion, chest tube drainage in addition to standard therapy was reported to result in more rapid discontinuation from mechanical ventilation [32]. Occasionally, increased resistance of venous return due to a large pleural effusion itself can result in IVC dilation.

### Parenchymal disease

#### Interstitial syndrome

**Fig. 7:**
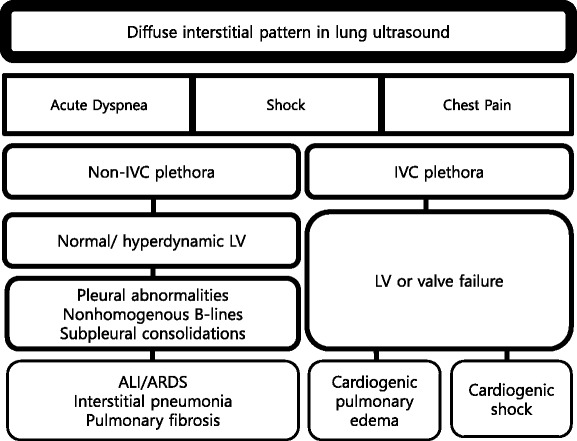
Algorithm for diffuse interstitial pattern in lung ultrasound. *IVC* inferior vena cava, *LV* left ventricle, *ALI* acute lung injury, *ARDS* acute respiratory distress syndrome

**Fig. 8:**
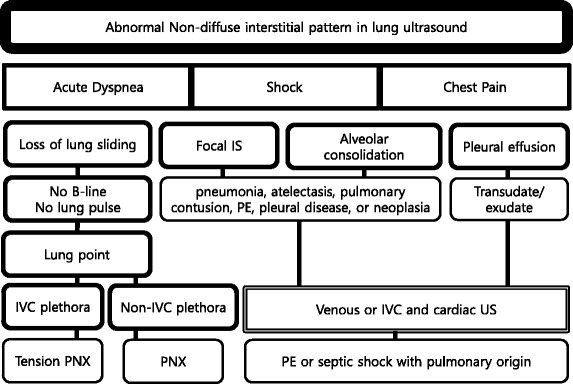
Algorithm for abnormal non-diffuse interstitial pattern in lung ultrasound. *IS* interstitial syndrome, *PE* pulmonary embolism, *IVC* inferior vena cava, *PNX* pneumothorax

Interstitial syndrome (IS) is divided into diffuse and focal patterns (Figs. 7 and 8). In diffuse IS, the posterior chest is not evaluated—only the eight anterolateral regions are examined [21]. Four regions per side (two anterior and two lateral) are evaluated. The anterior chest wall was delineated from the sternum to the anterior axillary line and was subdivided into upper and lower halves. The lateral zone was delineated from the anterior to the posterior axillary line and also was subdivided into upper and lower halves.

**Fig. 9:**
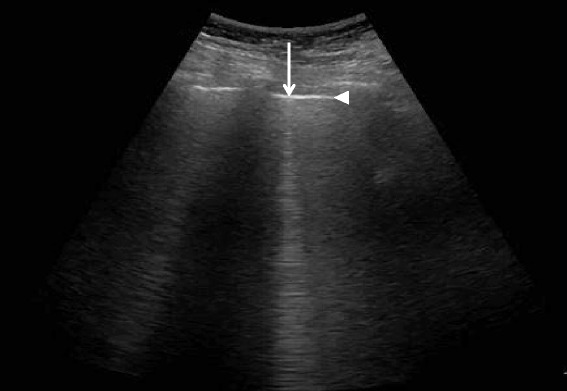
B-lines. B-line (*arrow*) is a bright comet-tail artifact that arises from the pleural line (*arrowhead*). It will move with lung sliding, if the sliding is present, and extends to the end of the screen without fading

Diffuse IS is defined as the presence of multiple diffuse bilateral B-lines with at least two positive scans on each side of the thorax (Fig. 9) [33]. Causes of diffuse IS include pulmonary edema of various causes, diffuse parenchymal lung disease (pulmonary fibrosis), or interstitial pneumonia [21]. The presence of diffuse bilateral B-lines has an 86–93 % sensitivity and 93–98 % specificity in the diagnosis of IS [33, 34]. Note that diffuse IS alone does not rule in any specific etiology: it could be detected in many dyspneic patients, as well as those presenting with shock and/or chest pain. As the circulatory and pulmonary systems are interconnected, an integrated MOPOCUS is mandatory. The presence of diffuse IS associated with either left ventricular (LV) systolic and/or diastolic dysfunction or valvular heart disease is highly indicative of cardiogenic pulmonary congestion [35]. Many recent studies have demonstrated the reliability of MOPOCUS as an approach to distinguish cardiogenic pulmonary edema from non-cardiogenic etiologies [2, 4, 5, 8, 9, 11, 14, 36]. Kajimoto et al. demonstrated that lung ultrasound alone showed a sensitivity and specificity of 96.0 and 54.0 %, respectively, for differentiating acute cardiogenic pulmonary edema from pulmonary disease, while lung-heart-IVC integrated ultrasound had a sensitivity and specificity of 94.3 and 91.9 %, respectively [2]. Generally, isolated ultrasonography of a single organ itself has low accuracy in differentiating acute heart failure from other causes of acute dyspnea. Acute dyspnea (clinical congestion) results from the failure of alveolar-capillary membrane (pulmonary congestion), which is induced by more stress, following the increase of LV-filling pressure (hemodynamic congestion) [35]. This is one good reason why lung ultrasound should be added to cardiac ultrasound. The presence of diffuse interstitial pattern associated to a normal heart indicates a non-cardiac cause of pulmonary edema, as acute lung injury (ALI)/ARDS, interstitial pneumonia, and diffuse parenchymal lung disease (pulmonary fibrosis, in a chronic setting). Unlike cardiogenic pulmonary edema, the associated lung findings for non-cardiac causes include pleural line abnormalities, non-homogenous distribution of B-lines, and subpleural echo-poor area (or consolidation) [21]. ARDS, in addition, has findings of spared area, loss, or reduced lung sliding and various consolidations [37].

If diffuse IS accompanies shock, the presumptive shock physiology is likely cardiogenic. The physician should try to elucidate the cause using IVC and cardiac ultrasound.

Focal (localized) interstitial sonographic pattern is seen in a variety of pathologies of pulmonary origin, such as pneumonia, atelectasis, pulmonary contusion, pulmonary infarction, pleural disease, or neoplasia [21]. Note that the main difference between diffuse and focal interstitial patterns on ultrasound is that the lung findings on the latter are asymmetrical. In itself, focal IS is not specific for an etiology: physicians need to integrate it in the entire clinical context, including other sonographic findings.

#### Alveolar consolidation

**Fig. 10:**
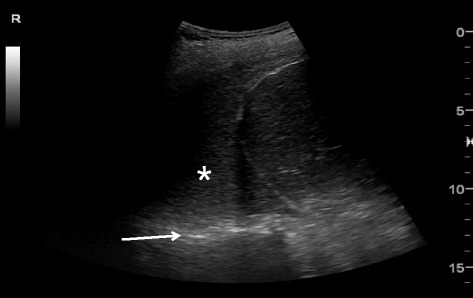
Lung consolidation. When the lung is consolidated (*asterisk*), it has a tissue-like appearance. The consolidation also allows penetration of the ultrasound beam, revealing the vertebral stripe (*arrow*)

**Fig. 11:**
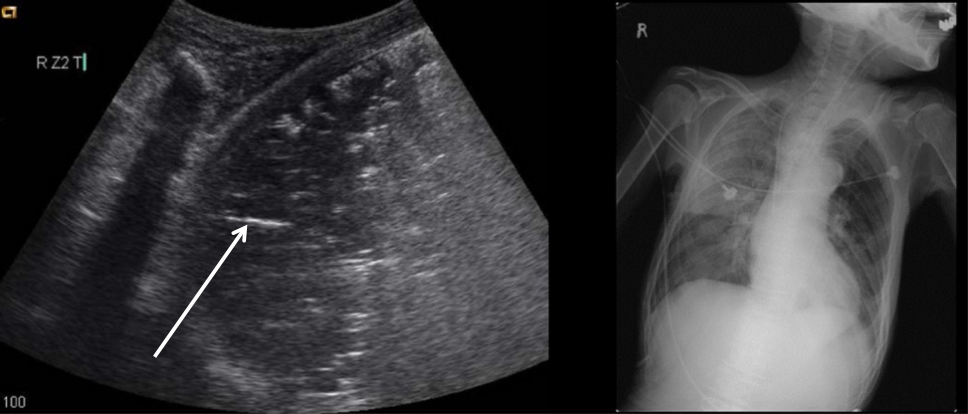
Alveolar consolidation and dynamic air bronchogram. Hypoechoic tissue-like patterned consolidation of the right upper lobe. Bright spots or streaky appearances are air bronchogram (*arrow*). A dynamic air bronchogram is visualized in the real-time image

The consolidated region of the lung is visualized as an echo-poor or tissue-like pattern, depending on the extent of aeration loss and fluid predominance (Fig. 10). A dynamic air bronchogram (Fig. 11) showing inspiratory centrifugal movement is a highly specific sign of pneumonia and is the most important sign to differentiate it from other causes of consolidation (atelectasis, pulmonary infarction, lung cancer) [38]. The alveolar consolidation pattern is usually associated with dyspnea or pleuritic chest pain [39]. In patients with hemodynamic instability, additional findings in MOPOCUS are needed to determine if the alveolar consolidation pattern results from pneumonia (septic shock) or PE.

### Inferior vena cava

**Fig. 12:**
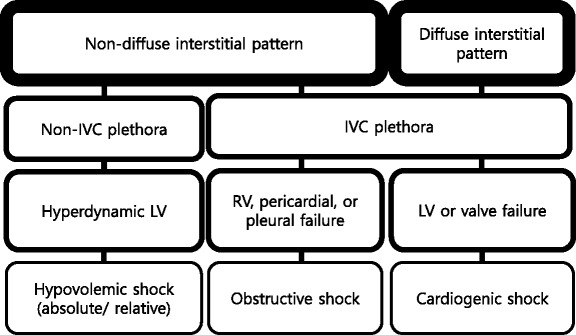
Algorithm for shock assessment. *IVC* inferior vena cava, *RV* right ventricle, *LV* left ventricle

**Fig. 13:**
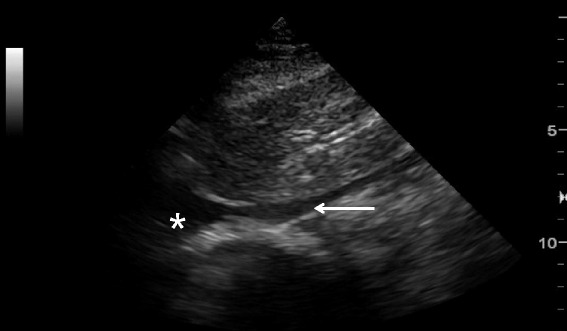
Inferior vena cava (IVC). IVC (*arrow*) draining into the right atrium (*asterisk*)

IVC ultrasound is particularly useful in shock assessment (Figs. 12 and 13). IVC is easily evaluated sonographically, using the liver as a window. Studies have examined the ability of IVC assessment to predict preload or volume responsibility: using IVC distensibility in patients with passive mechanical ventilation and maximal diameter of IVC or collapsibility in spontaneous breathing patients. Previous data on IVC distensibility in mechanically ventilated patients with sepsis provided encouraging results, being able to accurately predict volume responsiveness in sepsis or septic shock [40, 41]. However, recent studies, particularly those recruiting spontaneously breathing patients, have failed to show the same predictive value. Corl et al. found the collapsibility of IVC could not predict fluid responsiveness in a heterogeneous emergency department patient population with suspected hypovolemia [42]. In a practically time-limited clinical situation, the physician can evaluate this using other modalities. Most recent studies evaluating the effectiveness of MOPOCUS in undifferentiated shock use IVC size and respiratory variation as an indicator for fluid resuscitation [7, 10, 13]. While IVC size and variation in spontaneous breathing patients may serve as a surrogate for central venous pressure, it has not been proven a credible indicator of volume responsiveness on its own [43]. The approach using an integrated MOPOCUS assessment to guide fluid therapy needs further evidence. Combining lung ultrasound findings with IVC assessment, however, has a great potential to better inform fluid resuscitation decisions [44, 45]. Ultrasound findings of the absence of a diffuse interstitial pattern plus a small IVC diameter with high collapsibility of IVC indicate a fluid-tolerant state [22, 46]. If cardiac function is normal or hyperdynamic, as assessed using additional cardiac ultrasound, fluid boluses can be given, with serial clinical and sonographic reassessment [44, 45]. It is a decision-making process based on the concept of MOPOCUS and fluid tolerance. Furthermore, Caltabeloti et al. demonstrated the ability of lung ultrasound to define a fluid-tolerant state. In their study of patients with septic shock and ARDS whose LV ejection fraction (EF) was more than 50 % and pulmonary wedge pressure less than 18 mmHg, fluid loading produced only a transient improvement in hemodynamics and oxygenation, but aeration changes can be detected at the bedside lung ultrasound, which may serve as a safeguard against fluid over-resuscitation [47].

The presence of diffuse interstitial pattern with dilated and fixed IVC in shock patients prompts the physician to scan the heart, because the cause of shock is likely cardiogenic. Causes of obstructive shock (cardiac tamponade, tension pneumothorax, and PE) resulted in dilated IVC and non-diffuse interstitial pattern of the lung. A large pleural effusion resulting in diastolic failure or pulmonary hypertension caused by hypoxemia/hypercarbia also can lead to IVC plethora [31].

The key decisions for an acute care physician to make in undifferentiated shock depend on the categorization among three fluid management states: fluid resuscitate, fluid challenge, or fluid restrict. Using information from lung and IVC ultrasound, the physician can embark on an action and guide subsequent decision by cardiac ultrasound [46].

### Cardiac ultrasound

With information integrated from the preceding lung and IVC assessment, cardiac ultrasound can readily define the etiology of acute dyspnea and shock. It also plays a pivotal role in the case of visceral chest pain. This section describes the utility of cardiac ultrasound in the context of MOPOCUS for dyspnea, chest pain, and shock in turn.

#### Acute dyspnea

**Fig. 14:**
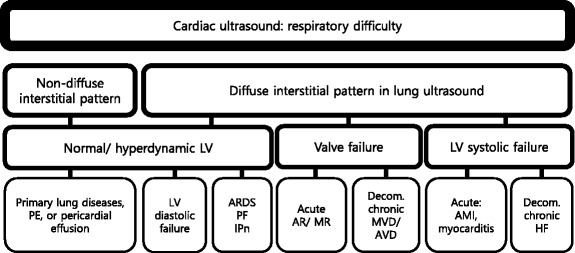
Cardiac ultrasound in respiratory difficulty. *PE* pulmonary embolism, *LV* left ventricle, *ARDS* acute respiratory distress syndrome, *PF* pulmonary fibrosis, *IPn* interstitial pneumonia, *AR* aortic regurgitation, *MR* mitral regurgitation, *Decom.* decompensated, *MVD* mitral valve disease, *AVD* aortic valve disease, *AMI* acute myocardial infarction, *HF* heart failure

**Fig. 15:**
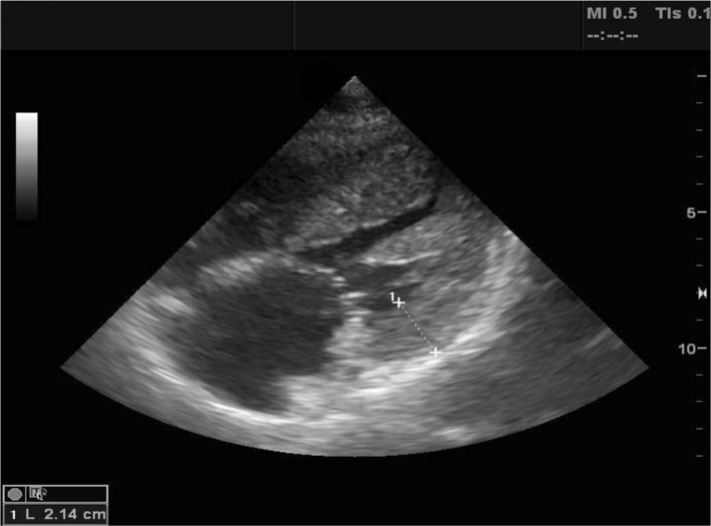
Left ventricular hypertrophy. Left ventricular hypertrophy involving both septal and lateral walls (2.14 cm). The left atrial appeared enlarged

Patient with diffuse interstitial pattern should have a focused cardiac ultrasound evaluation to determine the etiology, such as acute cardiogenic pulmonary edema, ARDS, or pulmonary fibrosis (Fig. 14). If LV systolic function is impaired, the most likely cause is cardiogenic pulmonary edema [2, 5, 9]. In the absence of gross signs of preexisting cardiac disease (i.e., LV enlargement or hypertrophy, right ventricular (RV) hypertrophy, or atrial dilation) (Fig. 15), the differentials can be narrowed down to acute processes, such as acute myocardial infarction or myocarditis [48]. Signs of preexisting cardiac disease are usually apparent in acute decompensation. If LV systolic function is normal, non-cardiogenic origin such as ARDS, interstitial pneumonia, or pulmonary fibrosis should be suspected, though cardiac pathologies such as significant mitral regurgitation (MR) or diastolic dysfunction are possible [2]. Significant valvulopathies can lead to cardiogenic pulmonary edema. The first task in valve evaluation is to exclude acute severe aortic or MR. Subsequently, the possibility of decompensated chronic severe aortic or MR/stenosis should be entertained [49]. Full evaluation with a comprehensive echocardiography is recommended for the quantitative analysis.

A non-diffuse interstitial pattern typically points to a pulmonary origin as a cause of dyspnea, in which lung ultrasound alone is usually sufficient.

#### Chest pain

**Fig. 16:**
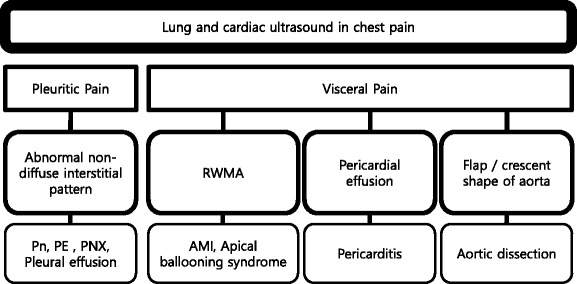
Cardiac ultrasound in chest pain. *RWMA* regional wall motion abnormality, *Pn* pneumonia, *PE* pulmonary embolism, *PNX* pneumothorax, *AMI* acute myocardial infarction

**Fig. 17:**
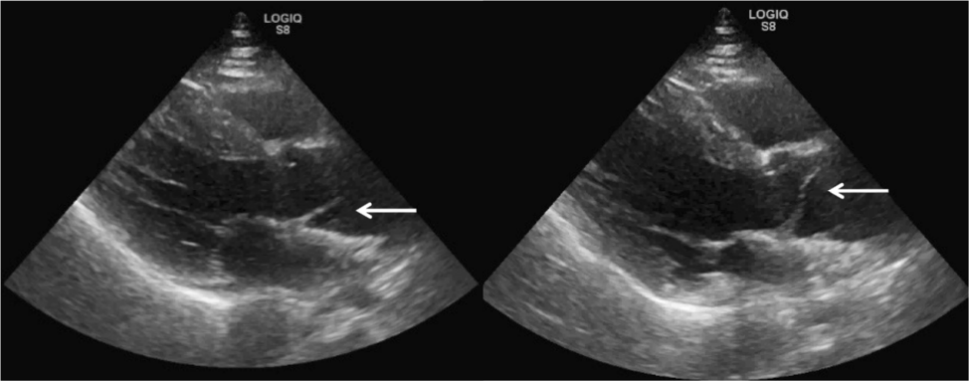
Thoracic aortic dissection. A moving intimal flap (*arrow*) in a proximal thoracic is visualized in the real-time image

**Fig. 18:**
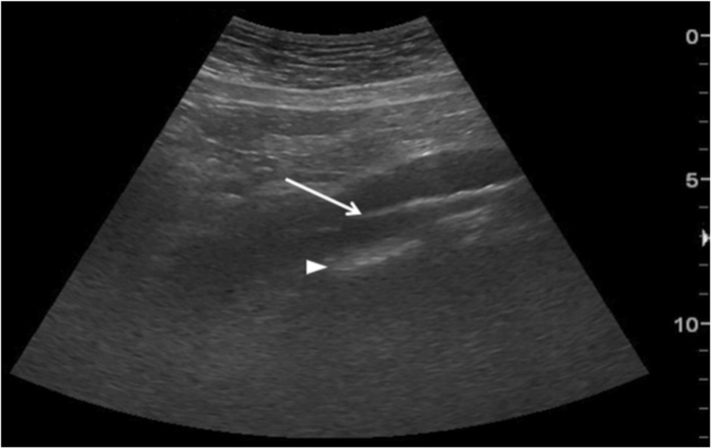
Abdominal aortic dissection. An intimal flap (*arrow*) dissecting into the lumen of the abdominal aorta. The *arrowhead* points to the vertebral stripe, on which the aorta lies

Pleural (pleuritic) chest pain results from lung pathologies such as pneumonia, pulmonary infarction, exudative pleural effusion, or pneumothorax (Fig. 16). These are readily diagnosed by lung ultrasound. On the other hand, visceral chest pain should prompt evaluation for acute coronary syndrome (ACS), pericarditis, or aortic dissection [50]. Following an initial electrocardiography (ECG), the presence of pericardial effusion, RV enlargement, or regional wall motion abnormality (RWMA) compatible to coronary artery distribution should be evaluated on cardiac ultrasound. Attempt should be made to visualize the thoracic aorta, starting from the aortic root, arch, and parts of the descending thoracic aorta behind the heart (Fig. 17). The abdominal aorta needs to be scanned when a dissection flap is visualized in the thorax above (Fig. 18). Note the multi-detector computerized tomography (CT) is the current gold standard in the evaluation for an aortic dissection. While the presence of RWMA in patients with ongoing chest pain without the previous history prompts appropriate management including percutaneous coronary intervention, absence of RWMA in patients with ongoing chest pain excludes a significant ACS [51]. Pericarditis is not always distinguished by clinical feature and ECG [52]. Cardiac ultrasound can be used as an adjunct, with supporting features such as the presence of a pericardial effusion and absence of RWMA. A flap in the aorta or a crescent shape of the aortic wall (direct sign) and aortic regurgitation, ascending aortic dilation, or pericardial effusion (indirect signs) suggest aortic dissection. They showed 98 % specificity for identifying patients with suspected type A aortic dissection combining aortic dissection risk score [53, 54].

#### Shock or shock-related symptoms or signs

**Fig. 19:**
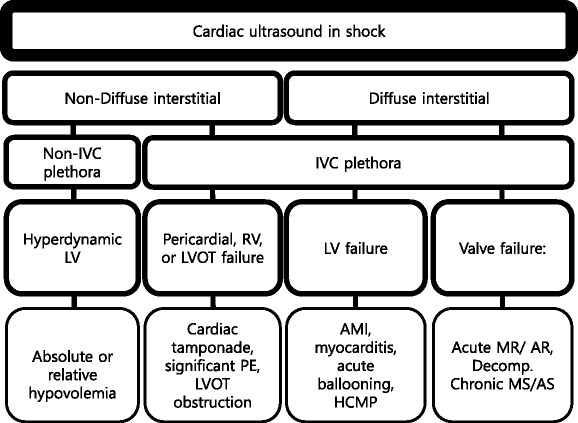
Cardiac ultrasound in shock. *IVC* inferior vena cava, *LV* left ventricle, *RV* right ventricle, *LVOT* left ventricular outflow tract, *PE* pulmonary embolism, *AMI* acute myocardial infarction, *HCMP* hypertrophic cardiomyopathy, *MR* mitral regurgitation, *AR* aortic regurgitation, *Decomp.* decompensated, *MS* mitral stenosis, *AS* aortic stenosis

Cardiac ultrasound in a shock patient provides critical information about the pericardium, bilateral chamber size and function, and valvular competency (Fig. 19). We emphasize that the priority is to rule out obstructive shock first, followed by cardiogenic shock, and then finally absolute or relative (distributive) hypovolemic shocks [49]. An approach based on the previous lung ultrasound pattern, diffuse interstitial pattern vs. non-diffuse interstitial pattern, is described here.

##### Non-diffuse interstitial pattern

It is suggestive of obstructive or hypovolemic shock: IVC plethora indicates obstructive shock, while a small non-plethoric IVC is usually associated with hypovolemic shock.

##### Pericardial failure (cardiac tamponade)

**Fig. 20:**
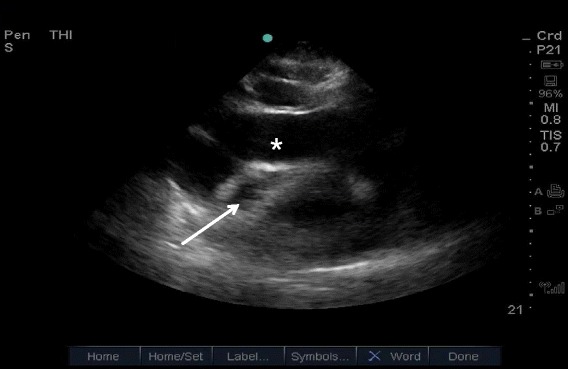
Cardiac tamponade. Right-sided heart chambers collapsed (*arrow*), due to increased intrapericardial pressure from a large pericardial effusion (*asterisk*)

**Fig. 21:**
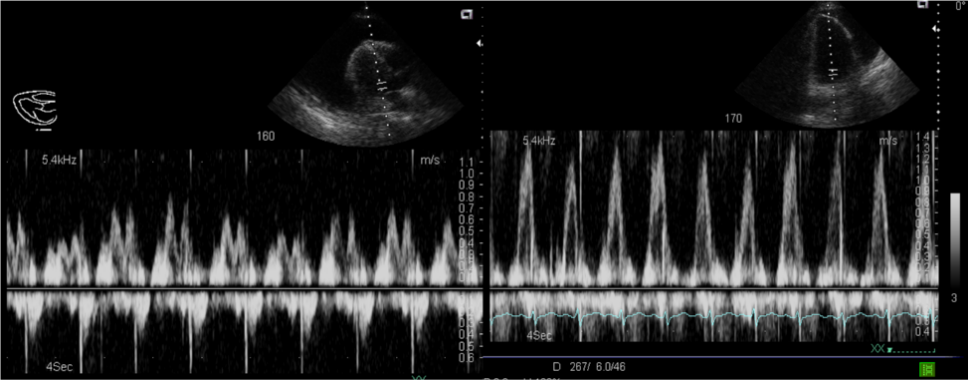
Cardiac tamponade physiology. (*Left*) Cardiac tamponade physiology, demonstrating reduced and aggravated variation of mitral valve inflow velocity. (*Right*) Post-pericardiocentesis: significant improvement in mitral valve inflow velocity

The sonographic signs of tamponade in the setting of a pericardial effusion include end-diastolic right atrium collapse (a highly sensitive sign) and RV collapse (less sensitive but more specific), IVC dilation, and greater than 25 % inspiratory variation in mitral inflow velocity measured by pulse-wave Doppler (Figs. 20 and 21) [55, 56]. In particular, IVC plethora (defined as a decrease in the proximal IVC diameter by <50 % during deep inspiration) has been described as the most sensitive (97 %) although least specific (40 %), while RV diastolic collapse is 48 % sensitive and 95 % specific [57]. It is important to remember that cardiac tamponade can complicate an aortic dissection or ACS (ventricular rupture); therefore, a high index of suspicion for two concurrent etiologies must be maintained [58].

##### RV failure (PE)

**Fig. 22:**
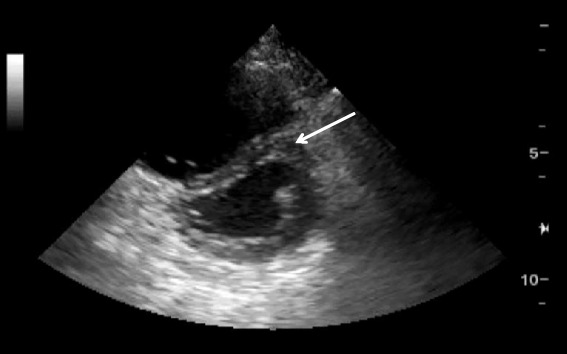
D-shaped left ventricle. The interventricular septum is normally round and bulges into the right ventricle (RV) throughout the cardiac cycle. Increased RV pressure causes the septum to be deformed to assume a “D”-shaped left ventricle (*arrow*)

**Fig. 23:**
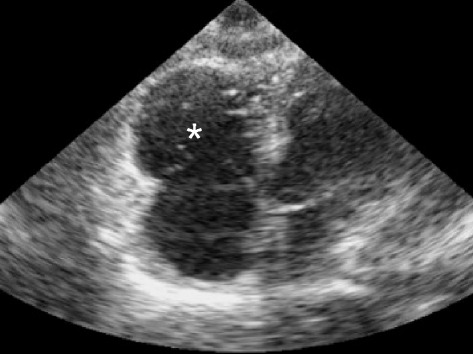
Pulmonary embolism, severe. Right ventricular enlargement (more than 0.9 of left ventricular size) is demonstrated (*white asterisk*). The RV free wall does not appear thickened, indicating an acute RV failure

Acute PE may lead to RV pressure overload and dysfunction (Fig. 22), which can be visualized by cardiac ultrasound. An RV-to-LV end-diastolic diameter ratio >0.9 was reported to indicate critical PE (Fig. 23) [59]. The absence of sonographic signs of RV overload or dysfunction practically excludes PE as the cause of hemodynamic instability [60]. Therefore, in a hemodynamically unstable patient with suspected PE, definite signs of RV pressure overload and dysfunction support emergency reperfusion treatment if immediate CT angiography is not feasible [61]. The potential pitfall in this setting is discriminating acute vs. chronic cor pulmonale. Chronic etiologies (COPD, chronic PE) can cause RV hypertrophy (diastolic RV thickness >6 mm) and beyond the values compatible with acute etiology (so-called 60/60 sign defined as RV acceleration time of <60 ms in the presence of tricuspid insufficiency pressure gradient <60 mmHg) [62, 63]. The physician keeps in mind that RV overload sometimes results from ARDS or RV infarction [64].

##### LV outflow tract failure

**Fig. 24:**
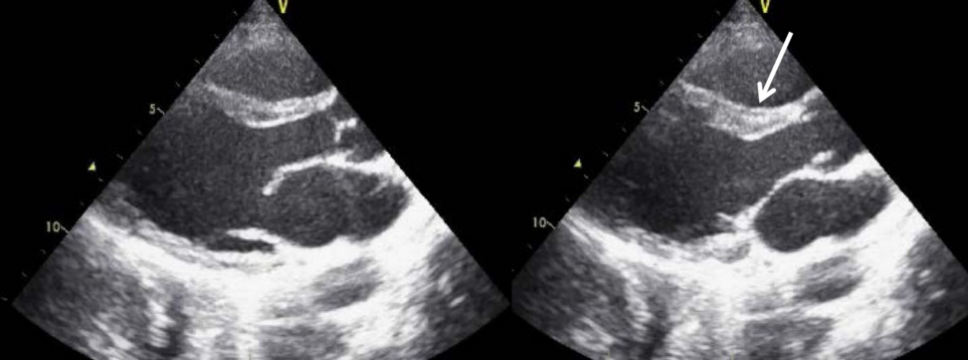
Apical ballooning syndrome. Severe hypokinesia of mid-ventricle sparing the basal segments (*arrow*). This is better appreciated during real-time scanning. Courtesy of Dr. Seong-Beom Oh

Dynamic LV outflow tract obstruction causing obstructive shock can be easily missed if cardiac ultrasound is not performed. Diagnosis of this is critical for the patient because the hemodynamic management is opposite to that of cardiogenic shock. Cardiac ultrasound generally shows hyperdynamic ventricular function with near complete or partial obliteration of the ventricular cavities. Additional sonographic signs include systolic anterior motion of the mitral valve, high ejection flow velocity in the LV outflow tract, and MR in the color Doppler image. LV outflow tract obstruction has been reported with LV hypertrophy, profound dehydration, excessive sympathetic stimulation, apical ballooning syndrome (i.e., takotsubo cardiomyopathy, Fig. 24), and acute myocardial infarction [65, 66]. Apical ballooning syndrome is reported to cause LV outflow tract obstruction in up to 25 % [67].

##### Other useful LV findings in shock states not caused by LV itself

After excluding obstructive shocks by information from sonographic findings of the lung, IVC, pericardium, and RV, the physician then needs to pay attention to the LV. Hyperdynamic LV without other abnormalities including significant valvular pathology suggests either distributive or hypovolemic shock. Distributive shock and hypovolemic shock commonly coexist in the critically ill, and early recognition and treatment with fluid resuscitation are paramount to manage these patients [49]. Therefore, we can practically categorize both as hypovolemic shock. It can be subdivided into absolute (hypovolemic) and relative (distributive) hypovolemic shock. Absolute hypovolemic shock has small-sized LV, while relative hypovolemic shock has normal-sized LV [31]. Jones et al. reported the presence of hyperdynamic left ventricular function (EF > 55 %) in emergency department patients with non-traumatic shock is highly specific for sepsis as the etiology of shock [68].

##### Diffuse interstitial pattern

Cardiogenic shock is most likely.

##### LV failure

MI with LV failure remains the most common cause of cardiogenic shock. The SHOCK trial registry demonstrated that predominant LV failure was the most common cause of cardiogenic shock, occurring in 78.5 % of patients. Patients with predominant LV failure complicating acute MI were more likely to have an anterior MI. Inferior MI was less often associated with LV failure but associated with a greater risk of mechanical complications [69]. Therefore, the presence of an extensive anterior MI or mechanical complications (severe MR due to papillary muscle rupture, ventricular septal defect, tamponade secondary to cardiac rupture, etc.) is a major concern in this setting [70]. In these settings, cardiac ultrasound is the investigation of choice. The clinical presentations of myopericarditis, apical ballooning syndrome, and hypertrophic cardiomyopathy can be similar to ACS and even cardiogenic shock. Sonographic findings of apical ballooning syndrome is a moderate-to-severe mid-ventricular dysfunction and apical akinesia with preserved basal function [71].

##### Valve failure

**Fig. 25:**
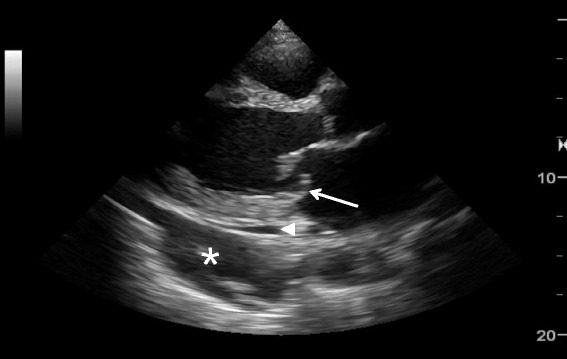
Flailed mitral valve. Flailed posterior mitral leaflet (*arrow*). Note the presence of a small pericardial effusion (*arrowhead*) and larger left pleural effusion (*asterisk*)

Valvular pathologies also are potential causes of cardiogenic shock (Fig. 25). The life-threatening acute severe regurgitation resulting from infectious endocarditis, acute myocardial infarction, or aortic dissection should be placed at the top of the list to be screened, as it prompts an emergent operation [70, 72]. Then hemodynamically compromised decompensation of preexisting aortic or mitral stenosis should be identified. In a patient suspected with aortic dissection complicating shock, not only severe acute aortic regurgitation but also pericardial effusion causing tamponade and acute myocardial infarction secondary to coronary artery involvement should be taken into account [73].

### Abdominal ultrasound

Abdominal ultrasound can help to determine the cause of hypovolemic (both absolute and relative) shock. Intra-abdominal source of blood loss or infection such as peritoneal effusion, ruptured abdominal aortic aneurysm or ectopic pregnancy, liver/spleen abscess, cholecystitis, cholangitis, or pyonephritis can be visualized [74].

## Conclusions

Multi-organ point-of-care ultrasound is a powerful adjunct to standard clinical assessment. It provides critical and timely information in the evaluation of patients presenting with acute dyspnea, chest pain, or shock: when and where it matters most, right at the bedside. It has become an indispensable part of the acute care physician’s armamentarium, in the battle for our patients’ lives.

## Abbreviations

ACS, acute coronary syndrome; ARDS, adult respiratory distress syndrome; COPD, chronic obstructive pulmonary disease; CT, computerized tomography; ECG, electrocardiography; FALLS, Fluid Administration Limited by Lung Sonography; IS, interstitial syndrome; IVC, inferior vena cava; LV, left ventricle or left ventricular; MOPOCUS, multi-organ point-of-care ultrasound; MR, mitral regurgitation; PE, pulmonary embolism; POCUS, point-of-care ultrasound; ROSC, return of spontaneous circulation; RV, right ventricle or right ventricular; RWMA, regional wall motion abnormality
